# The Missing Coronary: A Case Series of Inferior Wall Myocardial Infarction Due to Coronary Anomalies

**DOI:** 10.7759/cureus.65288

**Published:** 2024-07-24

**Authors:** Sriram Veeraraghavan, Bharath Raj Kidambi, Surendra K Naik, Ram Manohar Talupula, Soorampally Vijay, Amratansh Varshney, Abhilasha Munisingh

**Affiliations:** 1 Cardiology, SRM Medical College Hospital and Research Centre, Chennai, IND; 2 Cardiology, All India Institute of Medical Sciences, New Delhi, New Delhi, IND; 3 Cardiology, Al Dhannah Hospital, Abu Dhabi, ARE; 4 Cardiology, All India Institute of Medical Sciences, Raipur, Raipur, IND; 5 Interventional Cardiology, Aster Ramesh Hospitals, Vijayawada, IND; 6 Cardiology, Trilife Hospital, Bengaluru, IND

**Keywords:** inferior wall myocardial infarction (iwmi), coronary artery anomaly (caa), shark fin st-elevation ecg sign, judkins, right coronary artery anomaly

## Abstract

Coronary artery anomalies, while often asymptomatic, can sometimes present acutely in the context of myocardial infarction (MI). This case series highlights three unique instances of inferior wall MI precipitated by rare coronary anomalies. The first case involved a 40-year-old male with a congenital absence of the left circumflex artery, presenting with a “shark fin” ECG pattern in inferior leads. Urgent coronary angiography confirmed the anomaly and primary percutaneous coronary intervention (PCI) was performed on a superdominant right coronary artery (RCA). The second case details a 52-year-old male with a split RCA, initially undiagnosed due to apparently normal angiographic findings, later revealed to have a thrombotic occlusion of the posterior division. Careful re-evaluation and imaging from alternative angles facilitated successful PCI. The third case describes a 45-year-old male with an anomalous origin of the RCA from the left sinus of Valsalva, presenting difficulties during arterial engagement in PCI. A modified Judkins left catheter technique was employed to achieve selective cannulation and stent deployment. These cases underscore the importance of early recognition, accurate diagnosis, and innovative interventional strategies in managing acute MI due to congenital coronary anomalies.

## Introduction

Coronary artery anomalies represent a diverse spectrum of congenital abnormalities that can profoundly impact cardiovascular health. While many of these anomalies remain asymptomatic throughout life, some can present clinically, particularly in the context of acute coronary syndromes such as myocardial infarction (MI). The prevalence of coronary anomalies presenting as acute MI is estimated to be around 0.3% to 5.6% of all patients undergoing coronary angiography [1].

The absent left circumflex coronary artery (LCX) is a rare congenital anomaly that can predispose patients to acute coronary events. The pathogenesis of MI in absent LCX is multifactorial and can vary depending on the specific anatomical aberrations. Possible mechanisms include altered coronary circulation, with the right coronary artery (RCA) becoming superdominant to compensate for the areas typically supplied by the LCX. Additionally, the coronary steal phenomenon and coexisting atherosclerosis may also contribute to the increased risk of acute coronary events in these patients [2,3].

Another rare coronary anomaly is a split or duplicated RCA, where the single RCA trunk divides into two distinct branches. While this anomaly was previously considered benign, recent reports have suggested an increased risk of atherosclerosis and acute thrombotic occlusion of one of the split RCA divisions, leading to MI [4].

Another rare anomaly is the anomalous origin of the RCA from the left sinus of Valsalva. This anatomical variation can present unique interventional challenges when treating inferior wall MI. The anomalous course of the RCA may lead to difficulties in arterial cannulation and engagement during percutaneous coronary intervention (PCI), as the artery may take an atypical trajectory, making it harder to access and manipulate. Additionally, the altered blood flow dynamics and potential compression of the anomalous RCA during acute events can exacerbate the extent of myocardial injury and complicate the treatment approach [5].

This case series highlights the challenges faced in considering different etiologies, diagnostic methods, and treatment strategies while tackling coronary anomaly-related acute coronary syndromes.

## Case presentation

Case 1

A 40-year-old male with a history of hypertension presented to the emergency department with a sudden onset of severe chest pain radiating to the left arm and jaw, lasting for over 30 minutes. He also experienced giddiness with diaphoresis, nausea, and shortness of breath. On physical examination, the patient appeared in acute distress and was diaphoretic. His vital signs were notable for a blood pressure of 145/90 mmHg, heart rate of 35 beats/minute, respiratory rate of 32 breaths/minute, and oxygen saturation of 96% on room air. The cardiovascular examination revealed bradycardia, but the heart sounds were normal, with no jugular venous distension, murmurs, gallops, or rubs. The respiratory examination gave evidence of symmetric thorax; normal respiratory movements during breathing; no cyanosis, nasal flaring, or pursed lips; and no use of accessory muscles, wheeze, stridor, rales, rhonchi, or crackles.

**Figure 1:**
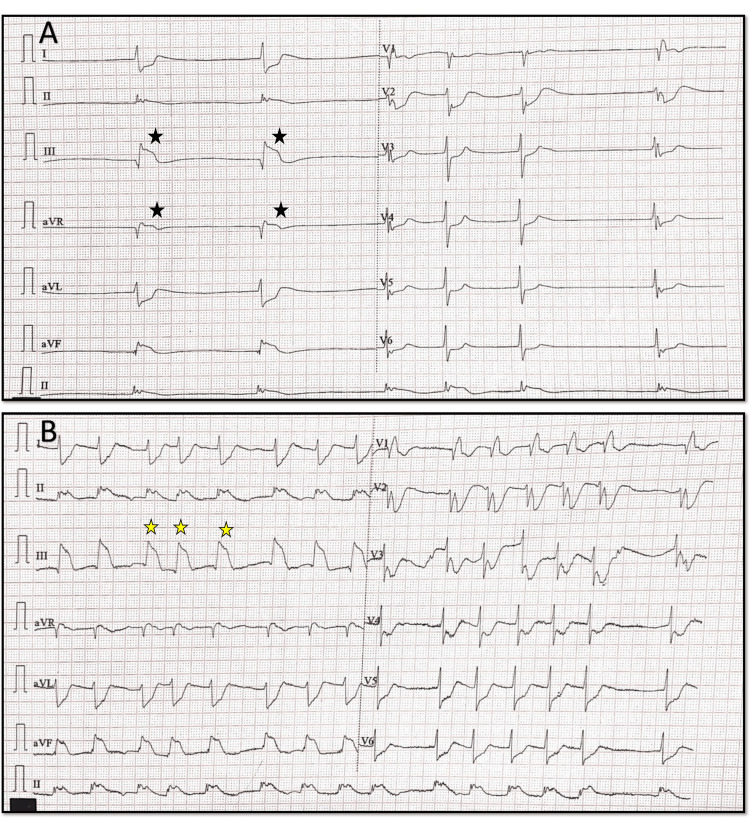
Initial 12 lead ECG before and after atropine. A, B: A 12-lead ECG showing ST elevation and bradycardia (black star), which improved after atropine was given in the next ECG. In B, the yellow star denotes the shark fin pattern characteristic of transmural involvement.

An ECG demonstrated junctional rhythm ST-segment elevation in leads II, III, and aVF, consistent with an inferior wall MI, along with reciprocal ST-segment depression in leads I and aVL, as well as significant ST depression in anterior precordial leads, suggesting the involvement of the posterior wall of the left ventricle. The heart rate improved after intravenous administration of atropine 0.6 mg, with distinctive ST-elevation patterns known as the “shark fin” pattern characteristic of transmural MI of a large territory (Figures 1A, 1B).

Laboratory tests revealed elevated cardiac biomarkers, with a troponin I level of 15 ng/mL and a creatine kinase-MB (CK-MB) level of 25 ng/mL. The patient’s lipid profile showed total cholesterol of 210 mg/dL, low-density lipoprotein of 130 mg/dL, high-density lipoprotein of 45 mg/dL, and triglycerides of 150 mg/dL. Initial management included administration of aspirin 325 mg, clopidogrel 600 mg loading dose, and an intravenous heparin bolus, followed by continuous infusion.

**Figure 2:**
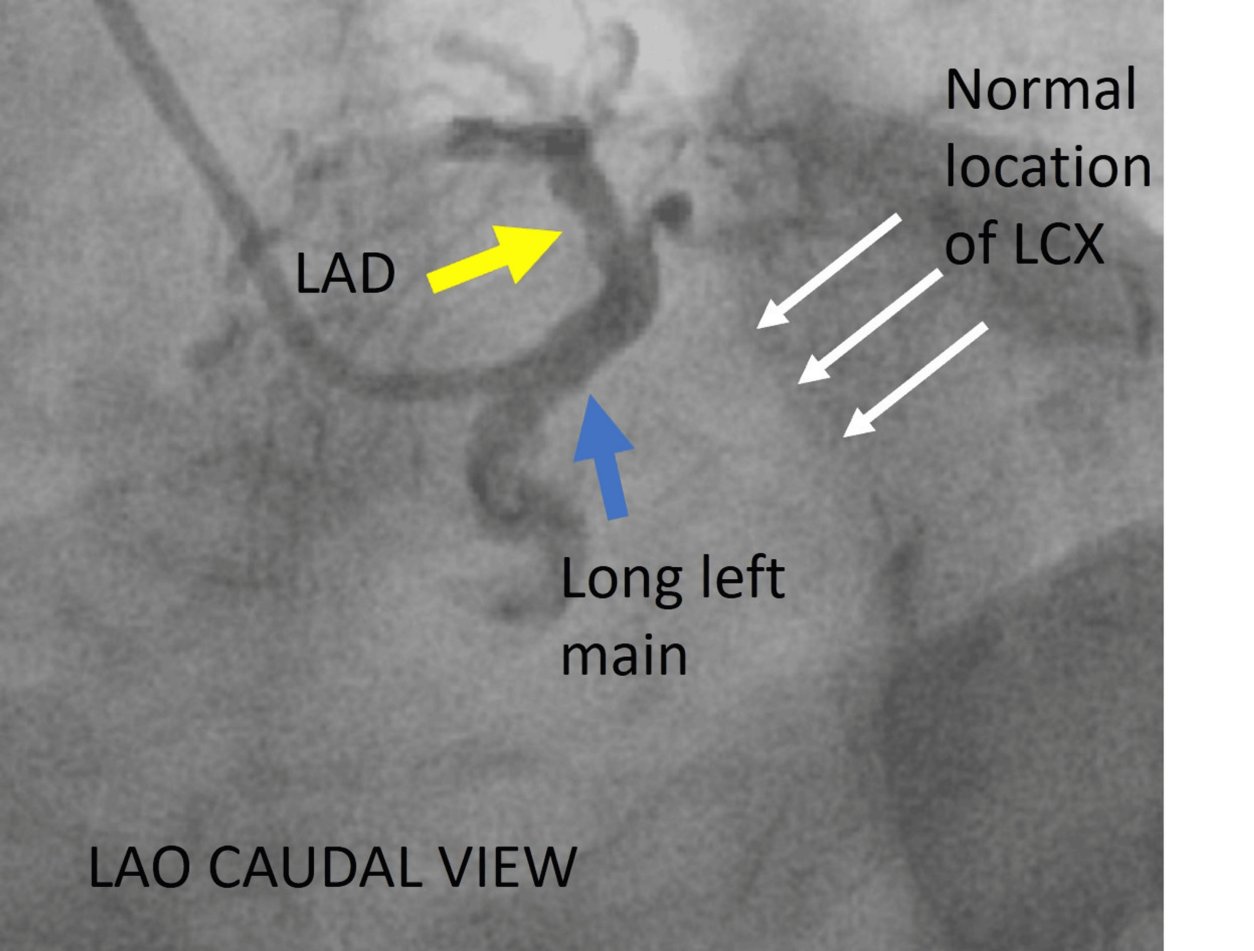
LAO caudal view left coronary injection. A: Cine angiography in the LAO caudal view showing the long left main (blue arrow), continuing as LAD (yellow arrow) with no vascular filling in the left atrioventricular groove, which is the usual location of LCX (white arrow). LAO = left anterior oblique; LAD = left anterior descending artery = LCX = left circumflex artery

**Figure 3:**
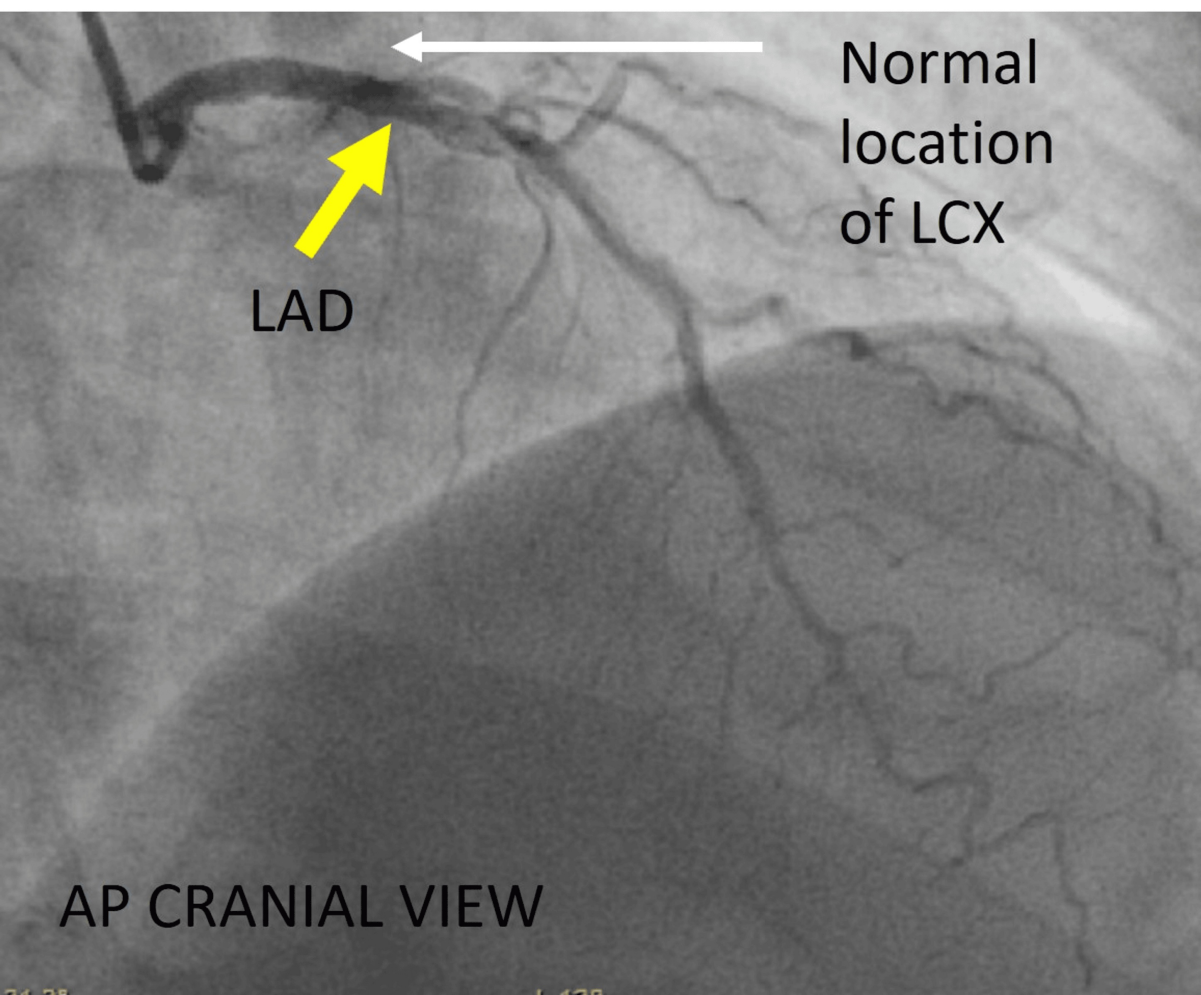
Different view of the left coronary system. Cine angiography in the AP cranial view showing LAD (yellow arrow) with no vascular filling in the left atrioventricular groove, which is the normal location of LCX (white arrow). AP = anteroposterior; LAD = left anterior descending artery; LCX = left circumflex artery

**Figure 4:**
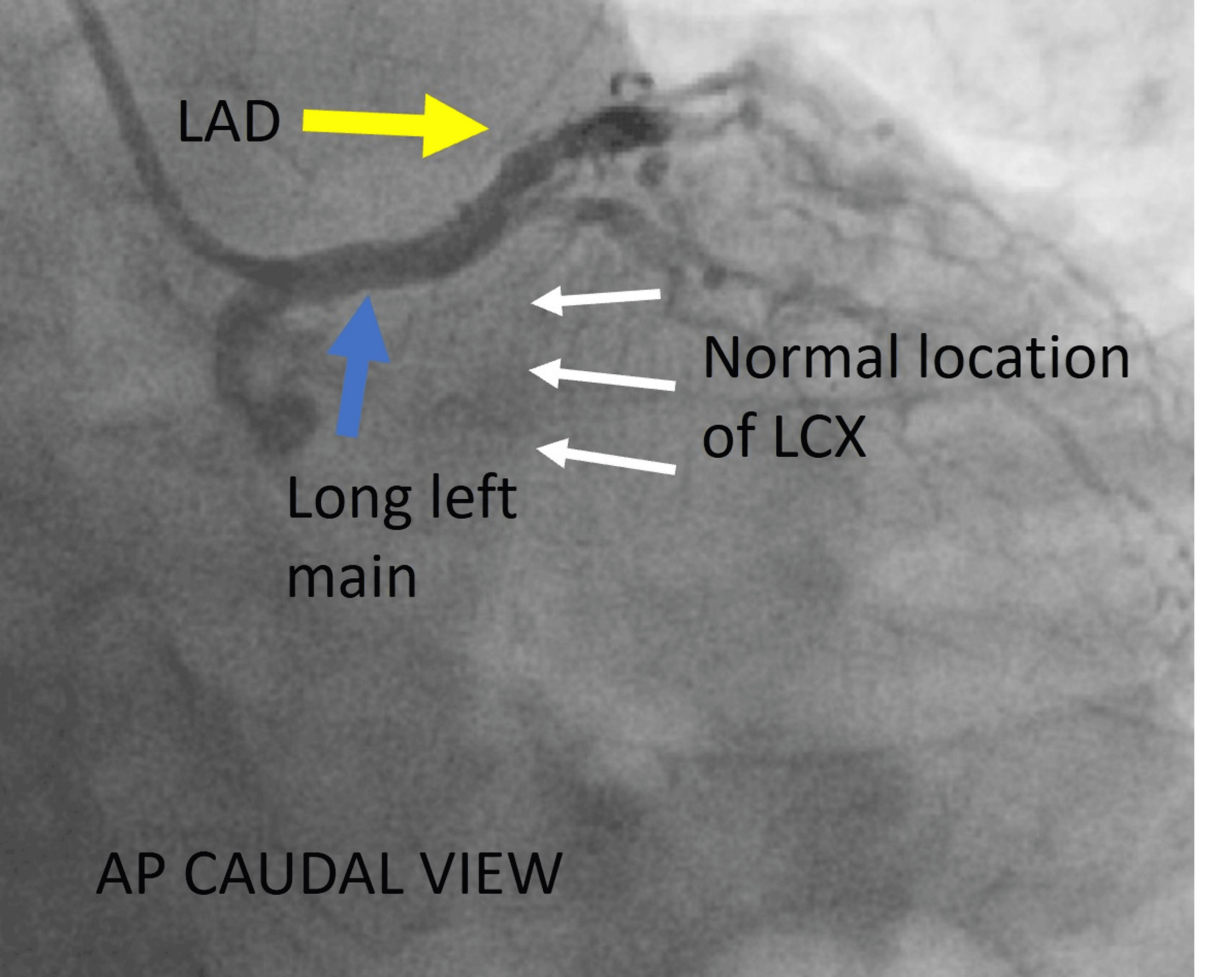
Another view of the left coronary system. Cine angiography in the AP caudal view showing the long left main (blue arrow), continuing as LAD (yellow arrow) with no vascular filling in the left atrioventricular groove, which is the normal location of LCX (white arrow). AP = anteroposterior; LAD = left anterior descending artery; LCX = left circumflex artery

Urgent coronary angiography was performed, revealing an unexpected finding. The left coronary injection showed a long left main artery that gave rise to the left anterior descending artery (LAD) only, with no identifiable LCX (Figures 2-4). There was no evidence of LCX arising from a separate ostium from the non-coronary or right coronary sinus.

**Figure 5:**
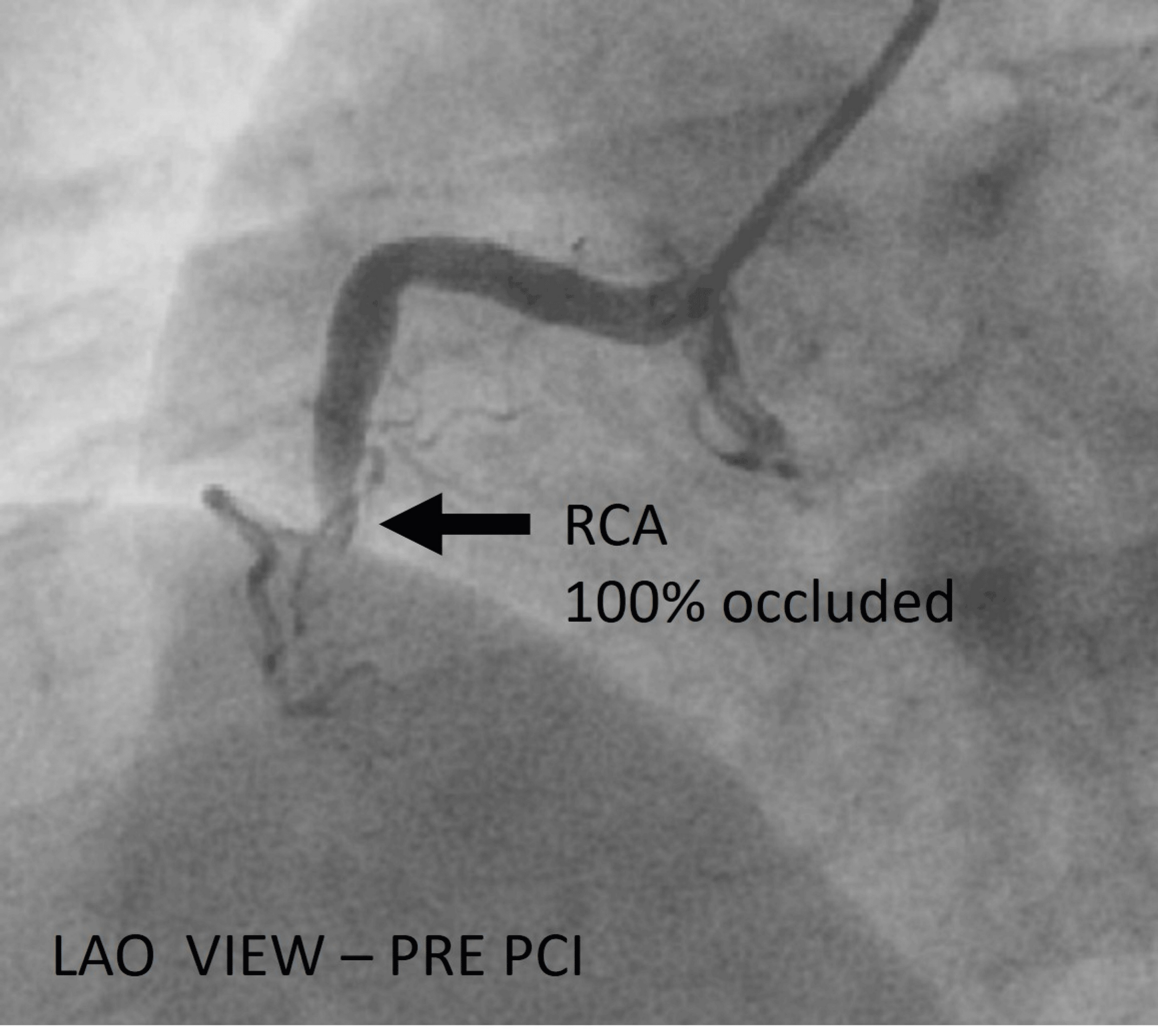
Angiography of the right coronary system. Cine angiography in the LAO view before PCI showing a completely occluded RCA (black arrow). LAO = left anterior oblique; RCA = right coronary artery; PCI = percutaneous coronary intervention

The RCA injection revealed a complete occlusion at the mid-segment just after a small acute marginal branch (Figure 5).

**Figure 6:**
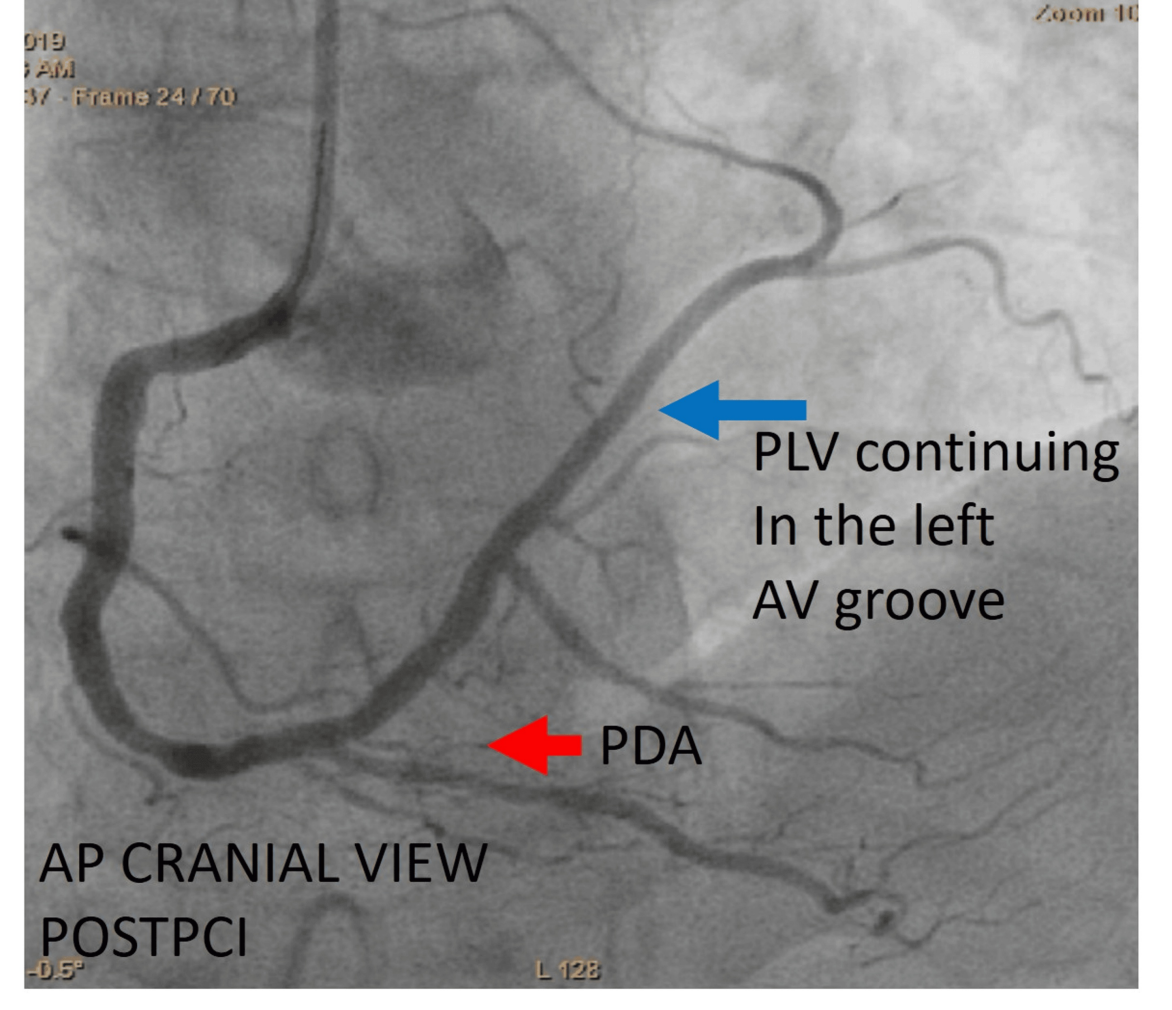
Post-angioplasty showing the full extent of arterial supply. Cine angiography in the AP cranial view after PCI showing RCA continuing after PDA (red arrow) as PLV (blue arrow) in the left atrioventricular groove supplying the LCX territory. AP = anteroposterior; PDA = posterior descending artery; PLV = posterolateral ventricular branch; PCI = percutaneous coronary intervention; LCX = left circumflex artery

**Figure 7:**
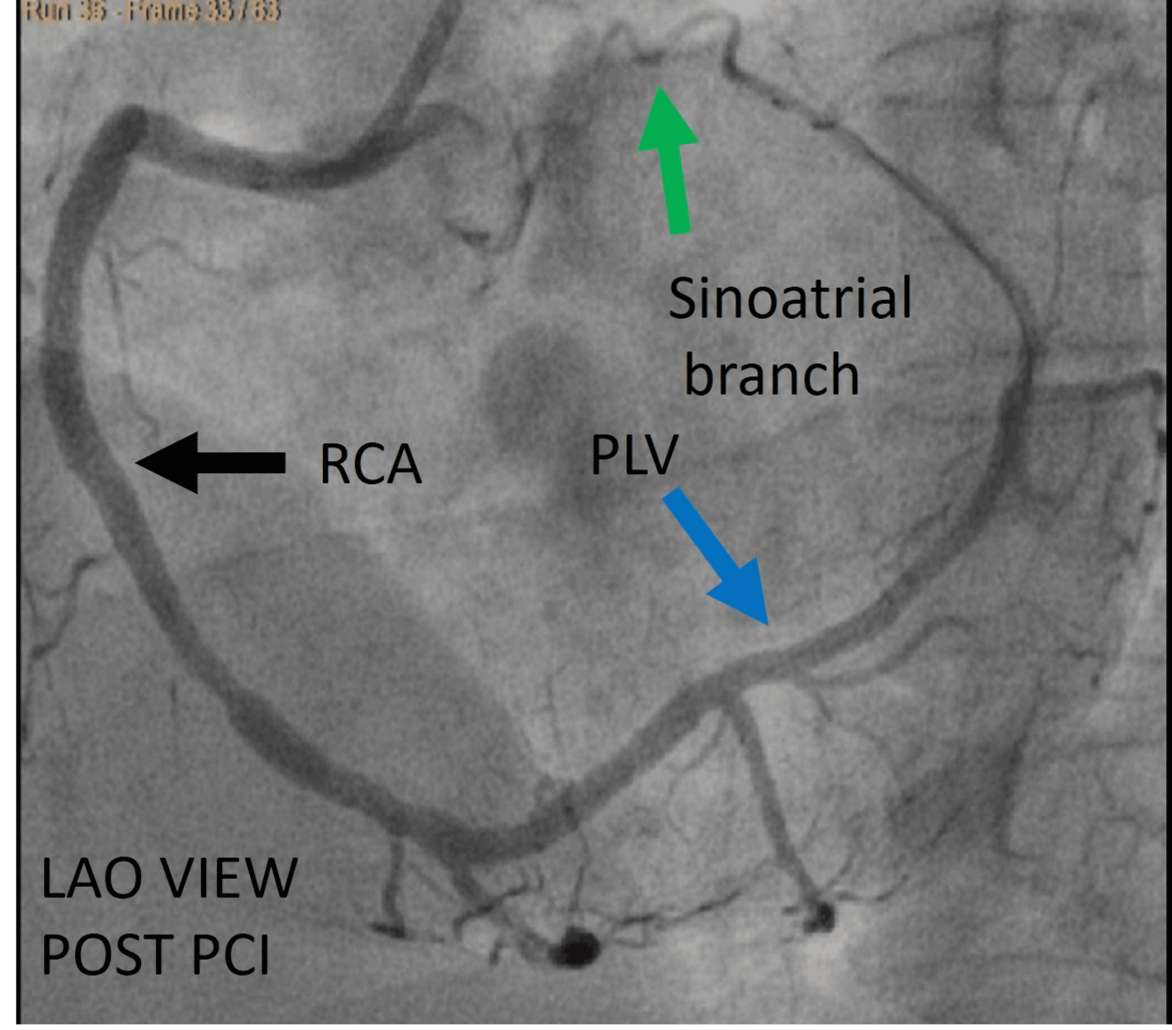
A different view post-angioplasty. Cine angiography in the LAO view after PCI showing RCA (black arrow), which was initially completely occluded, now continuing after PDA (red arrow) as PLV (blue arrow) in the left atrioventricular groove, which is the LCX territory, ending by giving rise to the sinoatrial branch (green arrow). LAO = left anterior oblique view; PCI = percutaneous coronary intervention; RCA = right coronary artery; PLV = posterolateral ventricular branches; LCX = left circumflex artery

The patient underwent primary PCI using a JR 3.5 catheter. A 0.014-inch BMW wire was carefully advanced to successfully cross the lesion. Subsequently, the lesion was pre-dilated using a 2.5 × 15 mm balloon to facilitate optimal stent deployment. Post-balloon dilatation, angiographic imaging revealed the distal RCA continuing as the posterior descending artery (PDA) and posterior left ventricular (PLV) artery, which extended along the left atrioventricular groove distally ending in the sinoatrial branch. A 3.0 × 28 mm drug-eluting stent was deployed across the lesion and post-dilated with a 3.5 x 15 mm non-compliant balloon to achieve optimal results and good distal flow. Post-PCI imaging confirmed that the RCA was super dominant, supplying the territories typically perfused by the LCX (Figures 6, 7).

**Figure 8:**
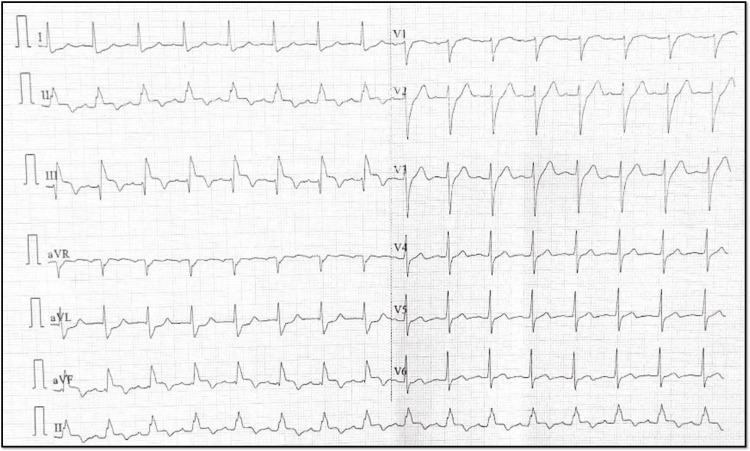
Post-percutaneous coronary intervention ECG. Post-procedure 12-lead ECG showing the resolution of the shark sign.

Following the procedure, the patient was monitored in the coronary care unit (CCU). Continuous telemetry showed no further arrhythmias and serial ECGs demonstrated partial resolution of the ST-segment elevation (Figure 8).

Troponin levels peaked at 35 ng/mL on day one post-MI and then gradually declined. The patient had an uneventful recovery and was discharged on day five with a prescription for aspirin 81 mg daily, clopidogrel 75 mg daily, atorvastatin 80 mg daily, metoprolol succinate 50 mg daily, lisinopril 10 mg daily, and continuation of amlodipine 5 mg daily for hypertension.

At discharge, the patient was scheduled for a follow-up appointment with cardiology in one week and referred to a cardiac rehabilitation program. Lifestyle modifications, including dietary changes, regular exercise, and stress management, were recommended.

Case 2

**Figure 9:**
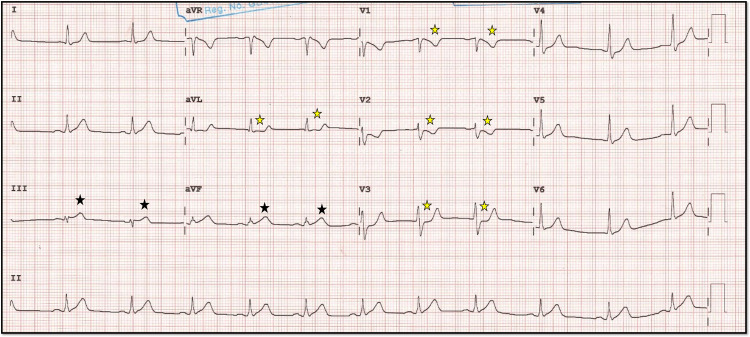
Initial ECG. The 12-lead ECG showing prominent T waves compared to ensuing R waves in inferior leads III, aVF (black star), with reciprocal ST depression in lead aVL and V1-V2-V3 (yellow star).

A 52-year-old male with a medical history significant for hypertension, type 2 diabetes mellitus, and a 20-year history of smoking presented to the emergency department with acute-onset chest pain radiating to his left arm, accompanied by diaphoresis and shortness of breath. The chest pain had been persistent for approximately four hours before his arrival. An ECG performed on admission revealed subtle ST elevation in inferior leads, with a disproportionately tall T compared to the R wave in lead III. There was subtle ST depression in lead I, aVL, V1, V2, V3, and T-wave inversion in V2 indicative of an inferior wall MI (Figure 9).

Transthoracic echocardiography demonstrated hypokinesia of the inferior wall with a moderately reduced left ventricular ejection fraction of 45%. Given the clinical presentation and imaging findings, the decision was made to proceed with urgent primary PCI.

**Figure 10:**
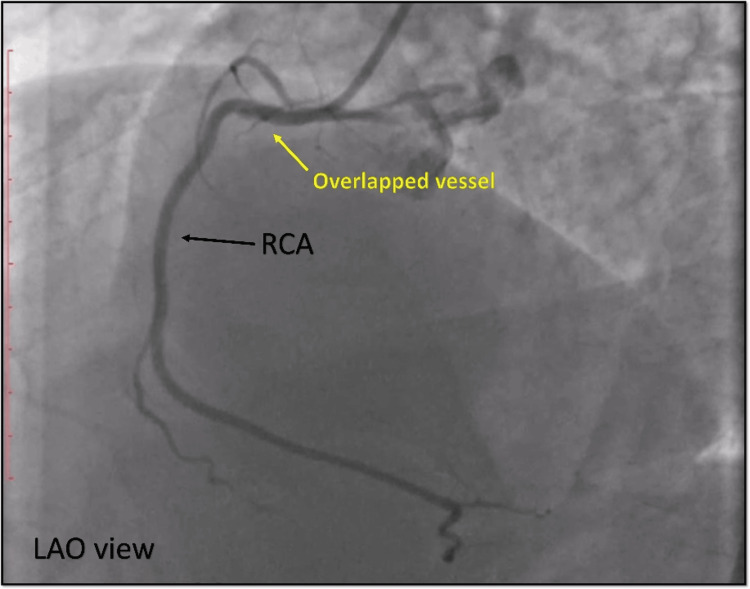
Diagnostic angiography. Cine angiography in the LAO view showing apparently normal RCA (black arrow); however, there is a hint of overlapped vessel (yellow arrow) in the proximal RCA. LAO = left anterior oblique view; RCA = right coronary artery

Coronary angiography was initially performed, revealing non-obstructive lesions in the LAD and LCX coronary arteries. The initial angiographic evaluation of the RCA showed a normal vessel with Thrombolysis in Myocardial Infarction grade III flow (Figure 10), which was perplexing given the patient’s clinical picture.

**Figure 11:**
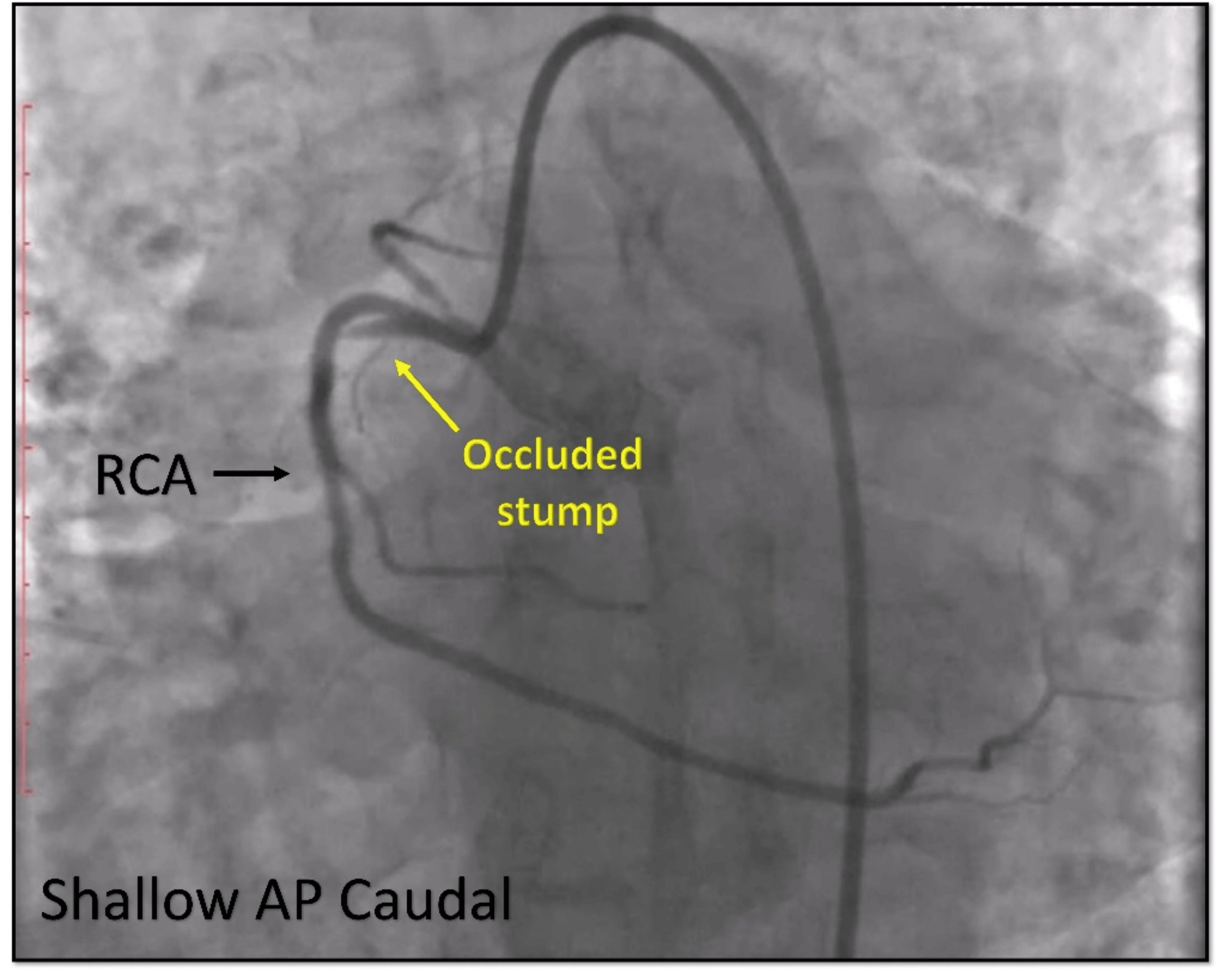
Angiography in a different view. Cine angiography in the shallow AP caudal view showing an occluded stump (yellow arrow) which appears to arise from the proximal portion of RCA (black arrow). AP = anteroposterior; RCA = right coronary artery

**Figure 12:**
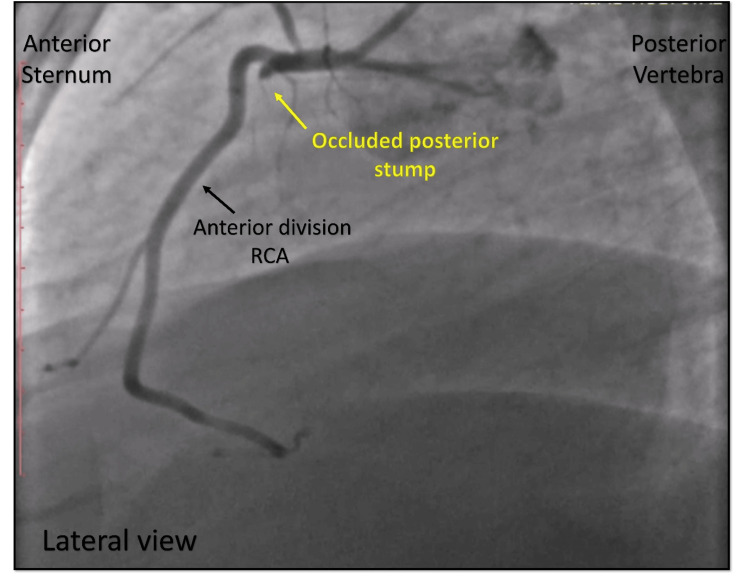
Lateral view of the same vessel. Cine angiography in the lateral view showing an occluded posterior stump of the split RCA (yellow arrow) delineated from the normal anterior division of the RCA (black arrow). RCA = right coronary artery

Upon further imaging from lateral and alternative views, a previously unnoticed abnormality was identified: a stump arising from the proximal segment of the RCA, exhibiting a complete thrombotic occlusion (Figures 11, 12). This structure was recognized as the posterior division of a bifurcated RCA, a rare congenital coronary anomaly.

**Figure 13:**
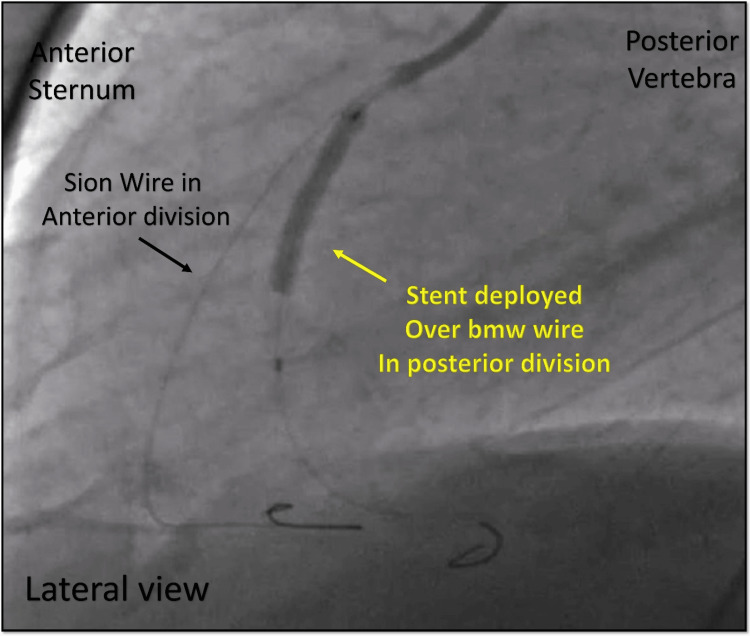
Angioplasty of the occluded vessel. Cine angiography in the lateral view showing the stent deployed over the BMW wire in the posterior division of the split RCA (yellow arrow) with another wire (SION) in the anterior division of the RCA (black arrow). BMW = balanced middle-weight; RCA = right coronary artery

**Figure 14:**
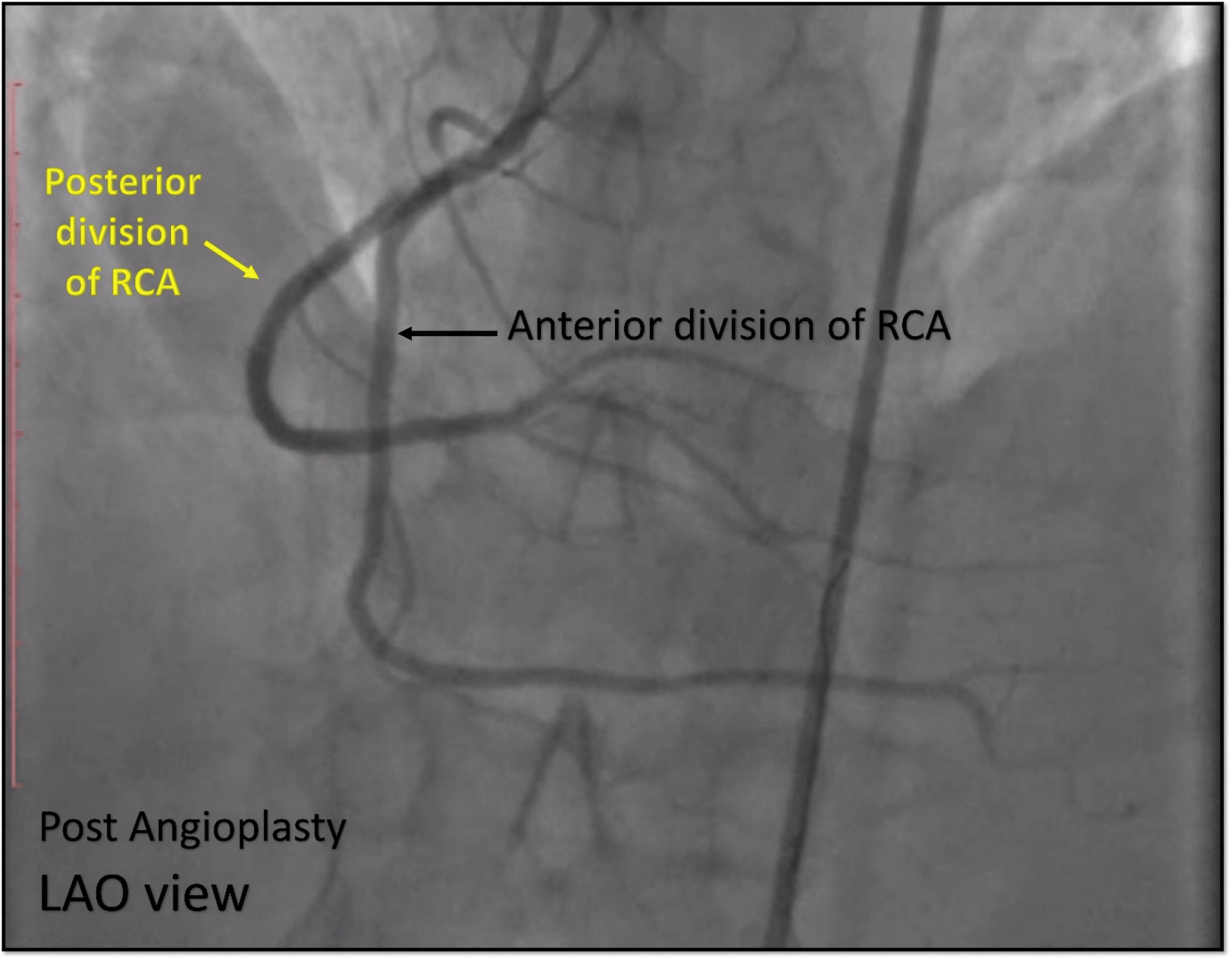
Post Angioplasty Cine angiography in the LAO view showing the stent deployed over the BMW wire in the posterior division of the split RCA (yellow arrow) with another wire (SION) in the anterior division of the RCA (black arrow). LAO = left anterior oblique view; RCA = right coronary artery; BMW = balanced middle-weight

**Figure 15:**
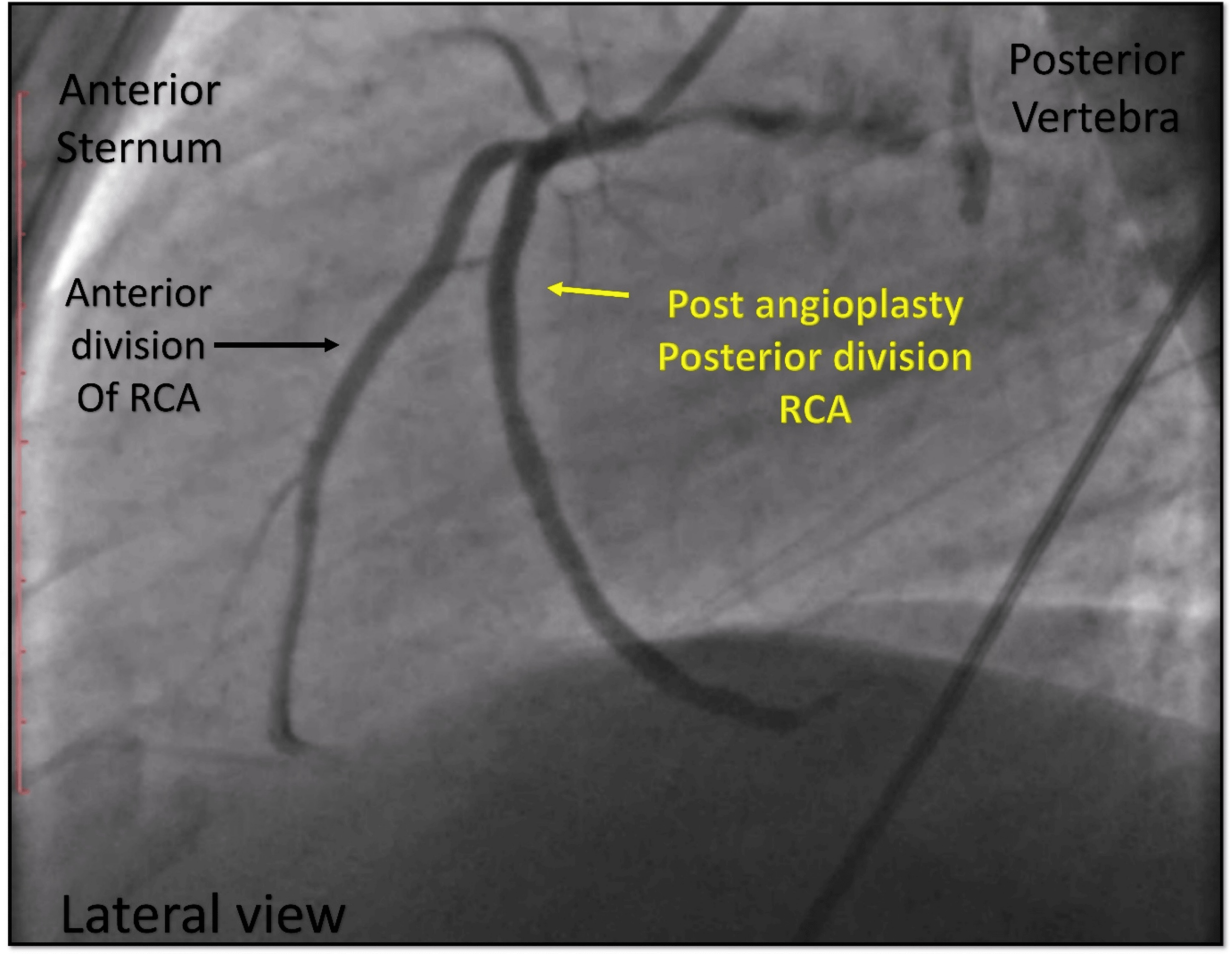
Post-angioplasty. Cine angiography in the lateral view showing good results after angioplasty, TIMI III flow in the posterior division of the RCA. TIMI = Thrombolysis in Myocardial Infarction; RCA = right coronary artery

Primary angioplasty was promptly performed on this occluded segment. The procedure involved careful navigation and crossing of the thrombus, followed by balloon angioplasty and subsequent stent deployment (Figures 13-15).

**Figure 16:**
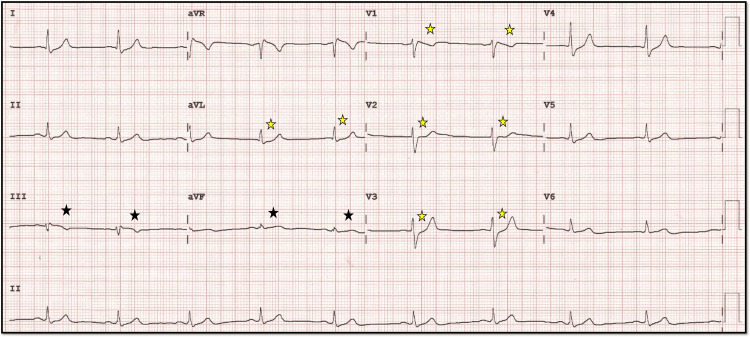
Post-angioplasty ECG. The 12-lead ECG following angioplasty showing the resolution of the prominent T waves in the inferior leads (black star) and the resolution of ST-segment to baseline in lead V1-V2-V3 (yellow star).

Post-procedure, the patient experienced significant relief from chest pain, and repeat ECG showed resolution of the ST-segment elevations (Figure 16).

The patient’s hospital course was uneventful, with no complications observed during his stay. He was started on appropriate secondary prevention measures including dual antiplatelet therapy, statins, beta-blockers, and angiotensin-converting enzyme inhibitors. He was discharged with a plan for close outpatient follow-up and lifestyle modifications aimed at addressing his cardiovascular risk factors.

Case 3

**Figure 17:**
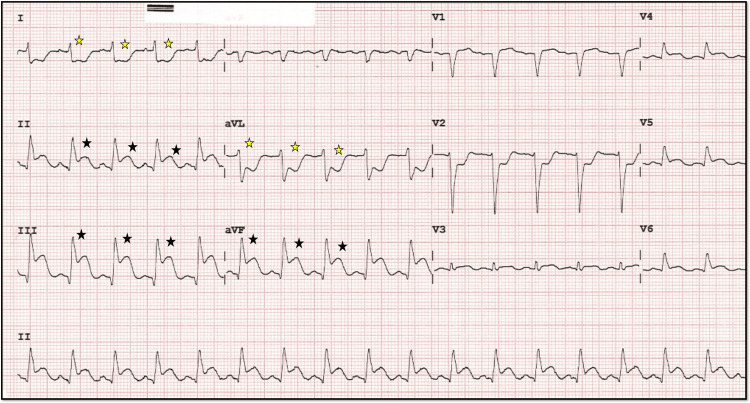
ECG on admission. The 12-lead ECG showing ST elevation in inferior leads II, III, aVF (black star) and the reciprocal ST depression in lead I, aVL (yellow star).

A 45-year-old male presented to the emergency department with sudden-onset, crushing chest pain that started about two hours before arrival. The patient reported experiencing severe, substernal chest pain that radiated to his left arm and jaw, accompanied by diaphoresis and nausea. The patient had no relieving factors, and the pain persisted despite taking over-the-counter medications. He had no significant past medical history and was not on any regular medications. On physical examination, the patient appeared in acute distress. Vital signs revealed a heart rate of 110 beats/minute, blood pressure of 95/60 mmHg, and a respiratory rate of 28 breaths/minute. The cardiovascular examination showed diminished heart sounds and no murmurs or gallops. Peripheral pulses were weak but palpable. The ECG showed ST-segment elevation in the inferior leads (II, III, aVF), consistent with an acute inferior wall MI (Figure 17).

Laboratory findings were significant for an elevated troponin I level of 8.2 ng/mL (reference range: 0-0.4 ng/mL) and CK-MB level of 80 U/L (reference range: 0-25 U/L). The complete blood count and basic metabolic panel were within normal limits.

**Figure 18:**
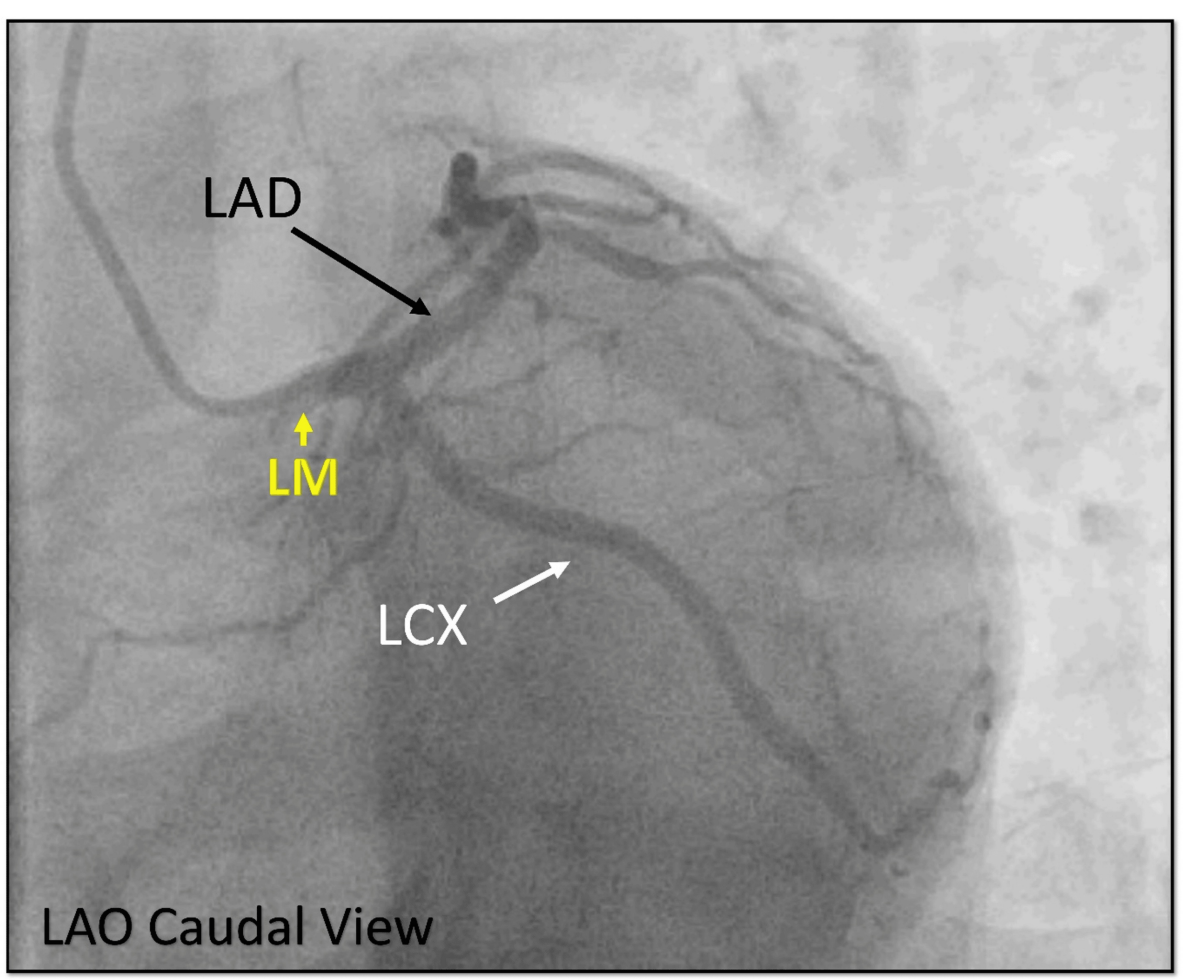
Diagnostic angiography. Cine angiography in the LAO view showing the normal division of the LM into the LAD and LCX. LAO = left anterior oblique; LM = left main; LAD = left anterior descending artery; LCX = left circumflex

He was shifted to the cardiac catheterization lab, and the initial diagnostic angiography revealed a normal left coronary system bifurcating into the LAD and LCX. The LCX was non-dominant without any disease (Figure 18).

**Figure 19:**
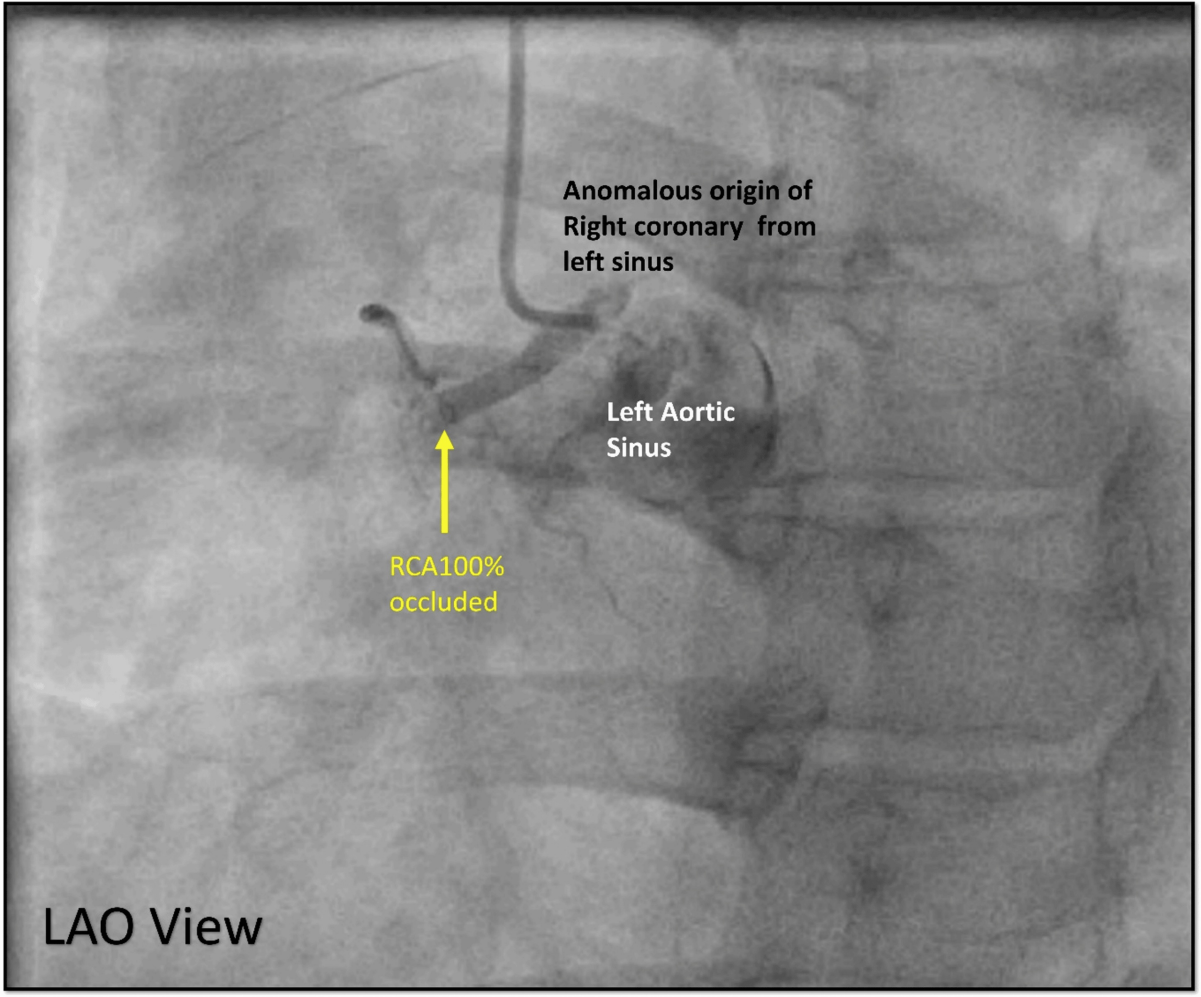
Right coronary shot. Cine angiography in the LAO view showing an anomalous origin of the RCA from the left sinus with complete occlusion of the proximal RCA (yellow arrow). LAO = left anterior oblique; RCA = right coronary artery

However, the RCA was not found in its usual location in the right sinus. Further investigation showed that the RCA was originating anomalously from the left coronary sinus, which was completely occluded in the proximal segment (Figure 19).

**Figure 20:**
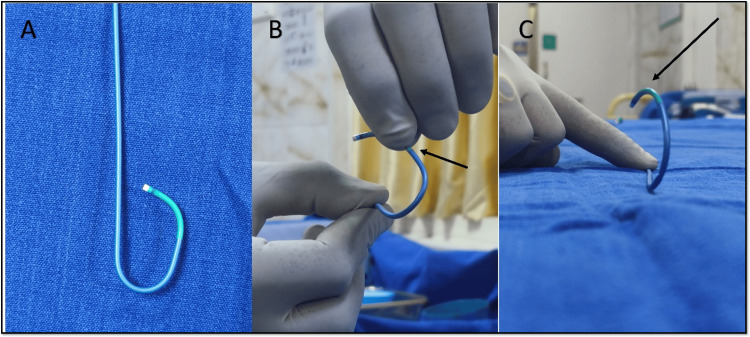
Modification of Judkins catheter ex-vivo. A: Normal Judkins left guiding catheter 3.5 with its intrinsic primary and secondary curve. B: Modifying the Judkins left guiding catheter, manually bending at 1.25 cm from the distal tip 90 degrees (black arrow) from its horizontal plane and holding it for a few minutes. C: Modified Judkins catheter showing the bend (black arrow) oriented with the anterior rightward origin of the anomalous origin of the right coronary artery ostium.

There was difficulty in engaging the right coronary sinus ostium with usual catheters (Judkins right (JR) 3.5) as the engagement angle differed. Engagement was also tried with an extra backup catheter (EBU 3.5) but could not achieve co-axial alignment. Finally, the Judkins left (JL 3.5), used for engaging the left system, was reshaped ex-vivo to engage the anomalous RCA ostium. This can be achieved by giving a 90-degree turn to the distal tip of the Judkins catheter, as shown in Figure 20. No additional heat source was required.

**Figure 21:**
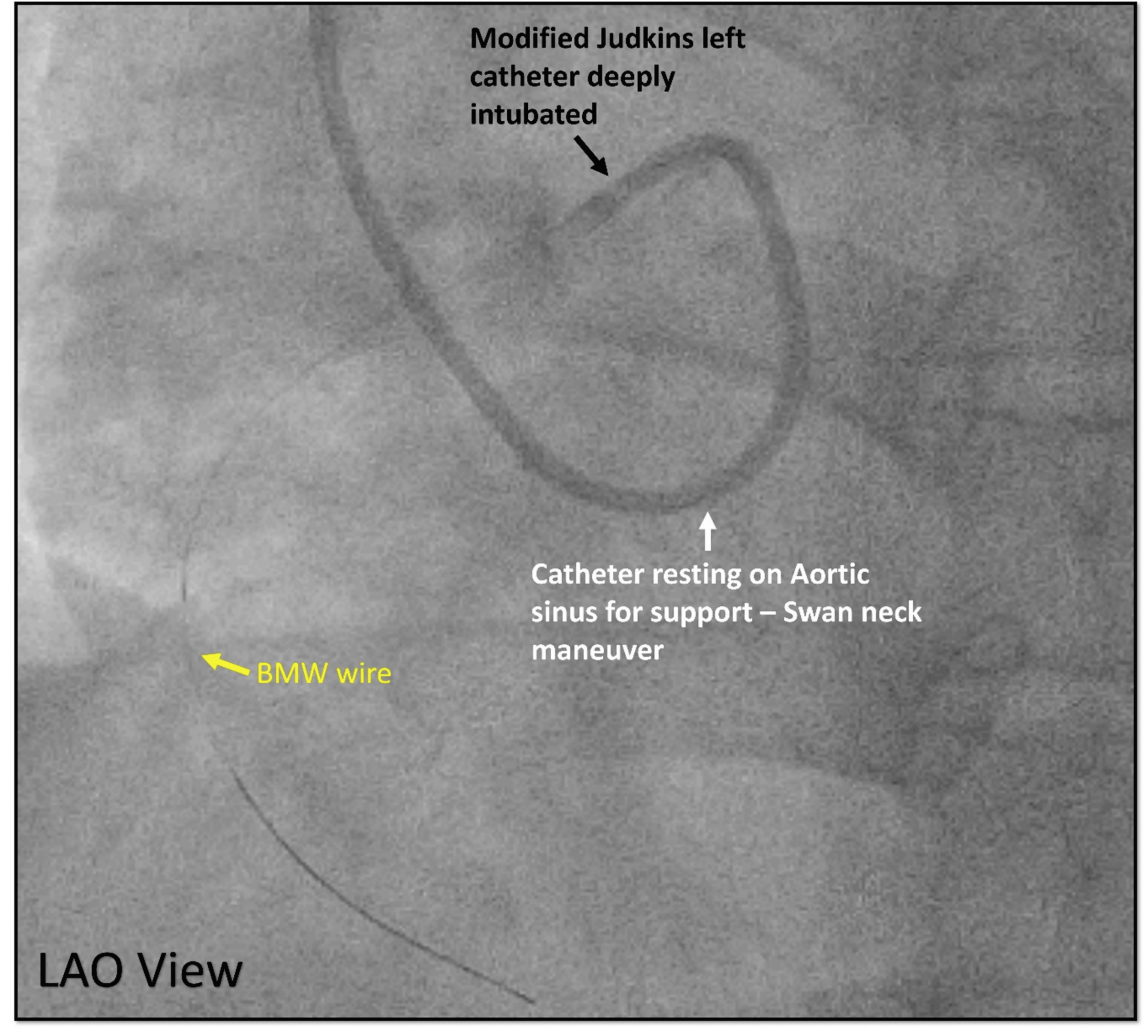
Engagement of the anomalous coronary. Cine angiography in the LAO view showing deep engagement of modified Judkins left (black arrow) with extra support from the aortic cusp (white arrow) helping to get a BMW wire across the lesion in the anomalous RCA (yellow wire). LAO = left anterior oblique view; RCA = right coronary artery; BMW = balanced middle-weight

After the engagement, the guiding catheter was pushed forward to get good support from the aortic cusps Swan neck maneuver (Figure 21).

**Figure 22:**
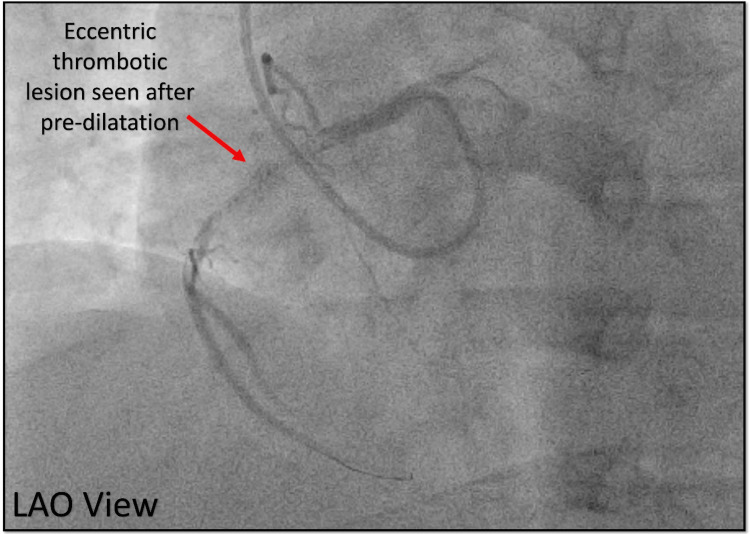
Diagnostic angiography after wire crossing the lesion. Cine angiography in the LAO view showing an eccentric thrombotic lesion (red arrow) in the RCA. LAO = left anterior oblique view; RCA = right coronary artery

**Figure 23:**
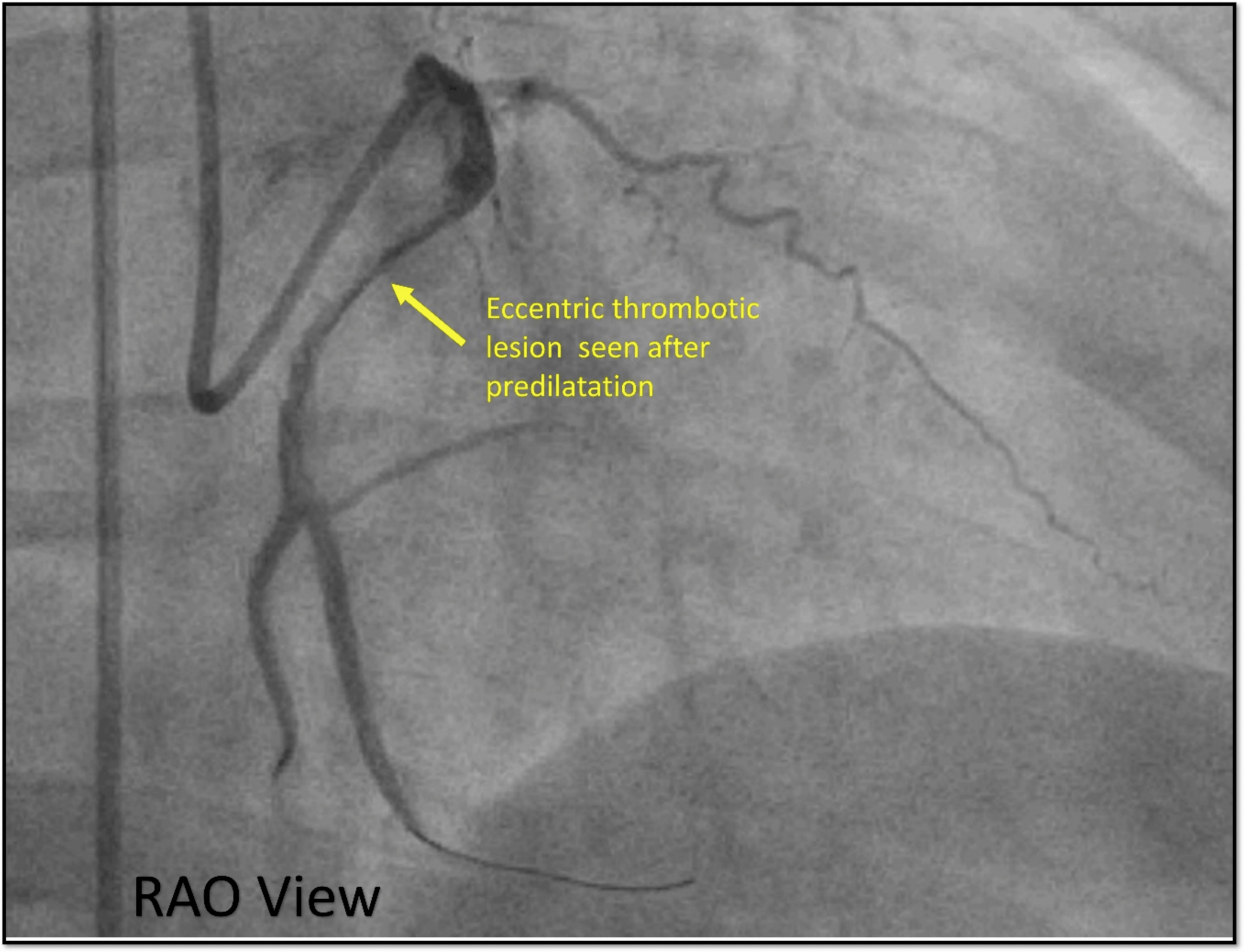
Orthogonal view RAO. Cine angiography in the RAO view showing the same eccentric significant thrombotic lesion in the RCA. RAO = right anterior oblique view; RCA = right coronary artery

After the successful engagement, a BMW wire was used to cross the lesion, predilated with an NC trek balloon (2.0 × 12 mm) which showed an eccentric thrombotic lesion in the proximal RCA confirmed in two different views (Figure 22, 23).

**Figure 24:**
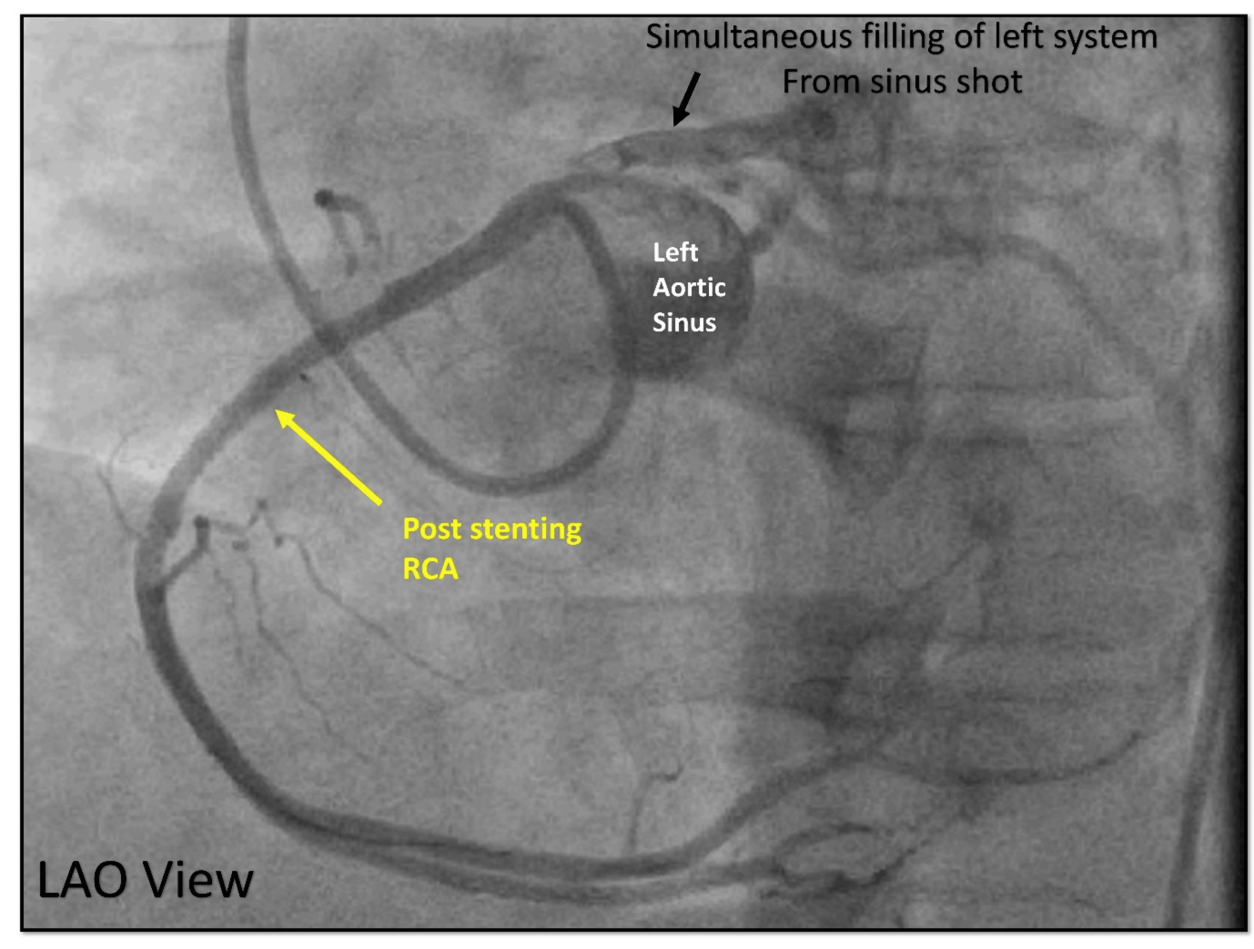
Final angiogram after angioplasty. Cine angiography in the LAO view, final shot after angioplasty (yellow arrow) showing good TIMI III flow in distal RCA. Simultaneous filling of the left coronary system (black arrow) from the left sinus shot is noticed. LAO = left anterior oblique view; TIMI = Thrombolysis in Myocardial Infarction; RCA = right coronary artery

A drug-eluting stent was deployed across the lesion resulting in a final good angiographic outcome (Figure 24).

The patient’s symptoms resolved after the successful primary PCI. He was monitored in the CCU and had an uneventful post-procedural course. The patient was discharged on appropriate medical therapy, including antiplatelet agents, beta-blockers, and statins, with instructions for cardiac rehabilitation and close follow-up.

## Discussion

In this case series, we presented three unique cases of inferior wall MI precipitated by rare congenital coronary artery anomalies. Early recognition and accurate diagnosis of these anomalies are crucial for effective management. Here, we discuss each case in detail, highlighting the diagnostic challenges and innovative interventional strategies employed.

Case 1: Congenital absence of the left circumflex artery

The first case involved a 40-year-old male presenting with the congenital absence of the LCX, evident from a “shark fin” ECG pattern in the inferior leads. This ECG finding indicated transmural involvement of a large myocardial territory, which typically suggests LAD territory infarctions. However, it pointed to a superdominant RCA as the culprit lesion in this context. The occlusion of a superdominant RCA effectively represents a double-vessel disease due to the extensive myocardial territory involved.

The congenital absence of the LCX is an uncommon anomaly, with a prevalence of only 0.003% to 0.067%. [6] Usually, the LCX originates as a branch of the left main coronary artery and enters the coronary sulcus or atrioventricular groove. In rare cases, the LCX fails to develop embryologically. This anomaly can lead to unnecessary delays during primary PCI if the operator searches for the missing artery. Recognizing the pattern of a long left main, the absence of the LCX in the left atrioventricular groove, and the absence of collaterals can suggest the congenital absence of the LCX, thereby preventing delays in management [6-8].

Case 2: Split right coronary artery

The second case involved a 52-year-old male with a split RCA, which initially presented a diagnostic challenge due to normal angiographic findings. This condition was later revealed to have a thrombotic occlusion of the posterior division. Split RCA is one of the most common coronary anomalies, with a prevalence of 1.23% [9]. This anomaly differs from a dual RCA, where the RCA arises from two separate ostial origins. A single RCA ostium splits early into two large branches in a split RCA.

In this case, the subtle ST elevation was due to one division being completely occluded while the other continued to supply a similar territory, dampening the ST elevation seen on the ECG. Proper diagnosis required detailed re-evaluation and alternative imaging angles. Viewing the anomaly from different angles and using a non-selective shot at the right sinus helped identify the occluded stump. Additionally, in scenarios involving ostial bifurcation disease, stenting one branch could potentially compromise the ostium of the other branch, complicating the procedure [9].

Case 3: Anomalous origin of the right coronary artery from the left sinus of Valsalva

The third case involved a 45-year-old male with an anomalous origin of the RCA from the left sinus of Valsalva. This condition occurs in 6% to 27% of patients with coronary anomalies [10]. In this case, the RCA path was anterior to the aorta without a slit-like ostium, which is considered safe. Proper assessment of the ostium required angiographic views from both the left anterior oblique and right anterior oblique angles.

Several angiographic factors influence guide catheter selection during PCI for anomalous RCA, including ostium configuration, aortic root dimensions, lesion location, and the type of hardware. Challenges in these interventions often arise from malalignment of the guide catheter with the artery’s initial course. In cases where the RCA ostium originates above the left sinus of Valsalva adjacent to the LCA, using an Amplatz left catheter with a larger secondary curve has proven effective. For the RCA ostium, which arises below the Sino tubular ridge, modified Judkins with a 90-degree bend can be used for adequate engagement. This technique, involving an anterior and cephalad pointing U-turn (“swan neck maneuver”), facilitates selective cannulation of the anomalous RCA and provides robust support for delivering angioplasty balloons and stents [11-14].

## Conclusions

Coronary anomalies, though rare, are a significant and intriguing differential diagnosis for ST-elevation MI. Employing various angulations during imaging can help identify these anomalous vessels, especially when uncertainty arises. Additionally, being prepared to modify catheters on the spot allows for better engagement of these challenging coronary anatomies, ultimately enhancing procedural success and patient outcomes.
